# Changes in male rat urinary protein profile during puberty: a pilot study

**DOI:** 10.1186/1756-0500-6-232

**Published:** 2013-06-15

**Authors:** Ariane Vettorazzi, Robin Wait, Judit Nagy, Jose Ignacio Monreal, Peter Mantle

**Affiliations:** 1 Department of Nutrition and Food Sciences, Physiology and Toxicology, Faculty of Pharmacy, University of Navarra, Irunlarrea 1, Pamplona E-31008, Spain; 2 Centre for Environmental Policy, Imperial College London, London SW7 2AZ, UK; 3 Faculty of Medicine Imperial College London, Kennedy Institute of Rheumatology, London W6 8LH, UK; 4 Imperial College London, Institute of Biomedical Engineering, London SW7 2AZ, UK; 5 Clinical Chemistry Department, University Clinic of Navarra, University of Navarra, Pamplona, Spain; 6 Current address: Department of Pharmacology and Toxicology, University of Navarra, Pamplona, Spain; 7 Current address: Kennedy Institute of Rheumatology, Nuffield Department of Rheumatology and Musculoskeletal Sciences, University of Oxford, London, UK

**Keywords:** Proteomics, PhastGel electrophoresis, Lipocalins, Protein droplet nephropathy, Ochratoxin A, Rat renal carcinogenesis, Androgen-dependent α2u-globulin

## Abstract

**Background:**

Androgen-dependent proteins (lipocalins) circulate in blood of male rats and mice and, being small (~ 18 kDa), pass freely into glomerular filtrate. Some are salvaged in proximal nephrons but some escape in urine. Several organic molecules can bind to these proteins causing, where salvage occurs, nephropathy including malignancy in renal cortex. In urine, both free lipocalins and ligands contribute to an increasingly-recognised vital biological role in social communication between adults, especially in the dark where reliance is on smell and taste. Crystal structure of the first-characterised lipocalin of male rats, α2u-globulin, has been determined and peptide sequences for others are available, but no study of occurrence during early puberty has been made. We have followed temporal occurrence in urine of juveniles (n = 3) for non-invasive pilot study by high resolution gradient mini-gel electrophoresis, tryptic digest of excised protein bands, and LC-MS/MS of digest to identify peptide fragments and assign to specific lipocalins. Study objective refers directly to external availability for social communication but also indirectly to indicate kinetics of circulating lipocalins to which some xenobiotics may bind and constitute determinants of renal disease.

**Results:**

Mini-gels revealed greater lipocalin complexity than hitherto recognised, possibly reflecting post-translational modifications. Earliest patterns comprised rat urinary protein 1, already evident in Sprague-Dawley and Wistar strains at 36 and 52 days, respectively. By 44 and 57 days major rat protein (α2u-globulin) occurred as the progressively more dominant protein, though as two forms with different electrophoretic mobility, characterised by seven peptide sequences. No significant change in urinary testosterone had occurred in Wistars when major rat protein became evident, but testosterone surged by 107 days concomitant with the marked abundance of excreted lipocalins.

**Conclusions:**

Qualitative temporal changes in the composition of excreted lipocalins early in puberty, and apparent increase in major urinary protein as two resolvable forms, should catalyse systematic non-invasive study of urinary lipocalin and testosterone dynamics from early age, to illuminate this aspect of laboratory rodent social physiology. It could also define the potential temporal onset of nephrotoxic ligand risk, applicable to young animals used as toxicological models.

## Background

Binding of pheromones to urinary proteins of rats and/or mice is increasingly becoming recognised as having complex biological roles in social communication and heterosexual interaction [1-3].

In the 1980s, search for organic substitutes for the lead tetra-ethyl added to the petrol of motor vehicles recognised several hydrocarbons of interest. However, toxicity tests revealed renal carcinogenic properties in male rats and further study showed a correlation with histopathological changes in kidney cortex. Hyaline droplets within nephron epithelia were attributed to accumulation of the putative carcinogen bound to a protein (α2u-globulin) the size of which allowed excretion in glomerular filtrate. Normal digestion of the protein in proximal tubule epithelia became inhibited when an isoprenoid hydrocarbon (e.g. d-limonene) is bound to the protein. The consequent intracellular accumulation of hyaline droplets caused epithelial cell necrosis and cell proliferation in response to injury. This non-genotoxic mechanism for rat renal carcinogenesis was a convenient finding, since attitude in human cancer risk assessment is less severe if a chemical carcinogen is shown to act *via* a mechanism that does not involve covalent binding to DNA [4].

α2u-globulin had long been known as a major urinary protein in male rats [5] and its role in rat experimental nephropathy was a timely finding in hydrocarbon toxicology. It was a topic of much toxicological discussion in the 1990s [6-9]. However, recent comprehensive review of the pathological connection between α2u-globulin nephropathy and renal tumours across many studied examples could not be so sure of a simple causal relationship [10].

The seminal studies of Roy et al. [11] resolved five isoelectric forms of α2u-globulin by 2-D electrophoresis and showed that the most prominent isoform with a pI of 6.1 was the first to appear in Fischer males at puberty (40 days old). Radioimmunoassay showed that, relative to total protein in liver, α2u-globulin ultimately rose to a maximum ~ 3.5 ng/mg at 70 days old. Subsequently, α2u-globulin was isolated from kidney cytosol of adult Fischer male rats and remained a single entity in SDS-PAGE and capillary electrophoresis [12], but occurred in greater concentration in kidney of rats given agents that induce α2u-globulin-nephropathy. However, α2u-globulin had already been described as resolvable electrophoretically into two distinct molecular forms [13].

All rats also produce another form of constitutive α2u-globulin, synthesised in the submaxillary gland *via* a set of genes that are different from those for the form in liver [14], but the salivary form is unlikely to complicate the picture in urine. The lipocalin is also found in lachrymal glands [15].

A possible extension of the range of hydrocarbons that can form biologically-active ligands with α2u-globulin to include ochratoxin A has been studied to determine whether protein droplet nephropathy is a prelude to the renal tumours that ochratoxin A can cause in male rats [16]. Clearly this nephropathy is not involved. However, binding of ochratoxin A to urinary α2u-globulin has since been seen [17] on PhastGel electrophoretograms (although with a 8-25% gradient), which could indicate potential for assisted nephron uptake of ochratoxin A during α2u-globulin salvage in proximal segments. Understanding the onset of such in young rats in toxicology research would be a factor in optimal experimental design. The crystal structure of α2u-globulin has been determined [18] and peptide sequences for others are available, but no study of occurrence during early puberty had yet been made.

For economic reasons experimental toxicology research often uses quite young rodents, in which toxicokinetics of xenobiotics may be influenced by small proteins circulating in blood. Currently there is only sparse data on this topic. The present pilot study has thus explored temporal changes in the small urinary protein pattern in two strains of laboratory rat during early puberty, collected without any stressful intervention, together with measurement of urinary testosterone as an indicator of expanding maleness. Several lipocalins have been partially characterised according to some of their peptide sequences after tryptic digestion.

## Results

**Figure 1:**
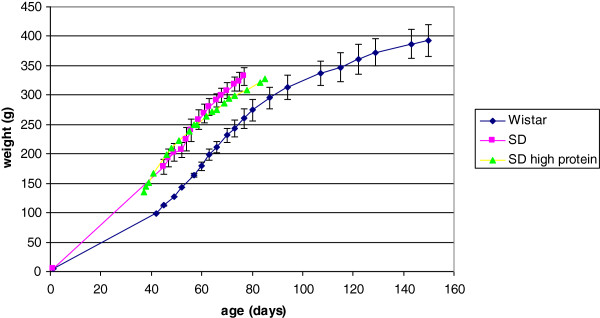
**Growth of groups of young male rats.** The Wistar group (n = 3) started with weight-matched individuals which were fed the maintenance diet. Sprague-Dawley (SD) groups (similar littermates, n = 3 or 4) were fed either a maintenance diet (14% protein) or a high protein (21%) formulation. Error bars for the SD high protein diet group have been omitted to avoid visual confusion in the graphic; the high protein diet appeared to promote a lower growth rate, evident after about 60 days of age (~250 g).

Growth of rats is illustrated in Figure 1. Comparison of Sprague-Dawley animals on maintenance and high protein diets showed slight rate divergence against the richer nutrition after about 9 weeks of age. However, animals from the high protein regimen were subsequently used for urinary protein characterisation to ensure that hepatic synthesis of lipocalins was not constrained by shortage of dietary nitrogen that might have occurred in rapidly growing juveniles on maintenance diet.

**Figure 2:**
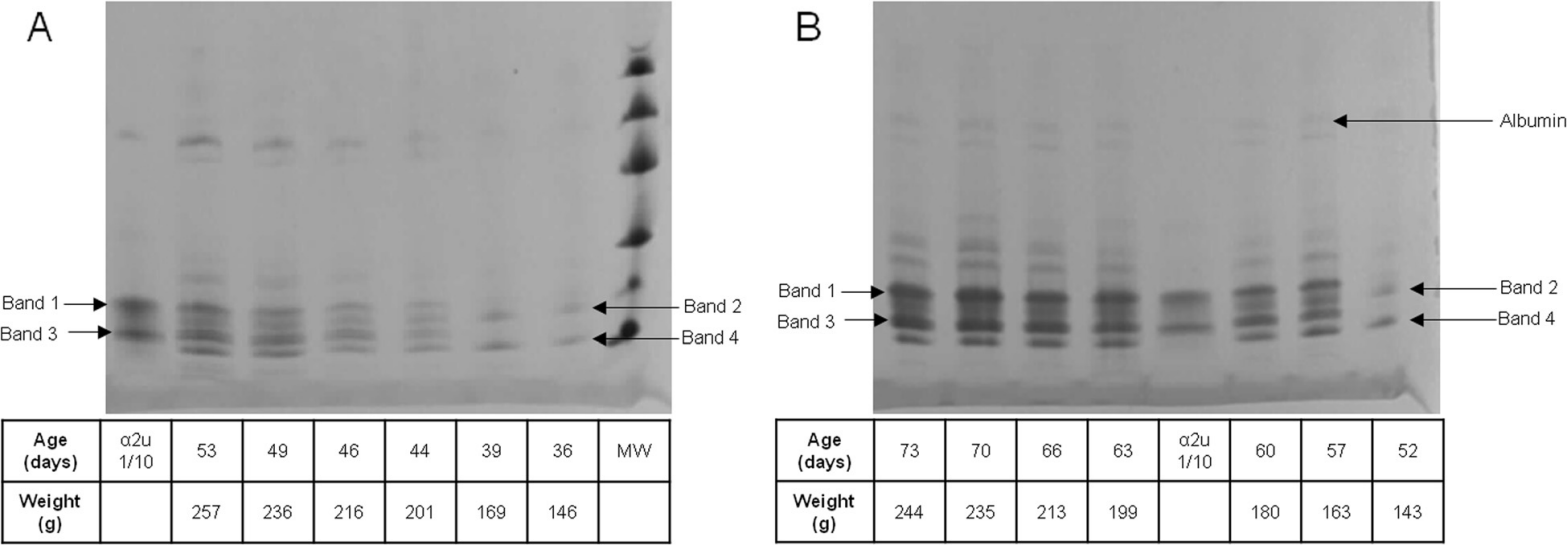
**A, Gel of Sprague-Dawley urines from increasing age (right to left) showing, in the 14-20 kDa region, transition from an initial pair of bands to a group of four in which two attributed to α2u-globulin become dominant (see also Figure **3**).** A band attributed to an albumin, by analogy with that in Figure 4B indicated (arrow), in the 45-66 kDa region apparently increased with age. **B**, Gel of Wistar urines from increasing age (right to left) showing a pattern analogous to that in Sprague-Dawley rats (Figure 4A). A band indicated by the arrow was identified from tryptic digest data as an albumin.

**Figure 3:**
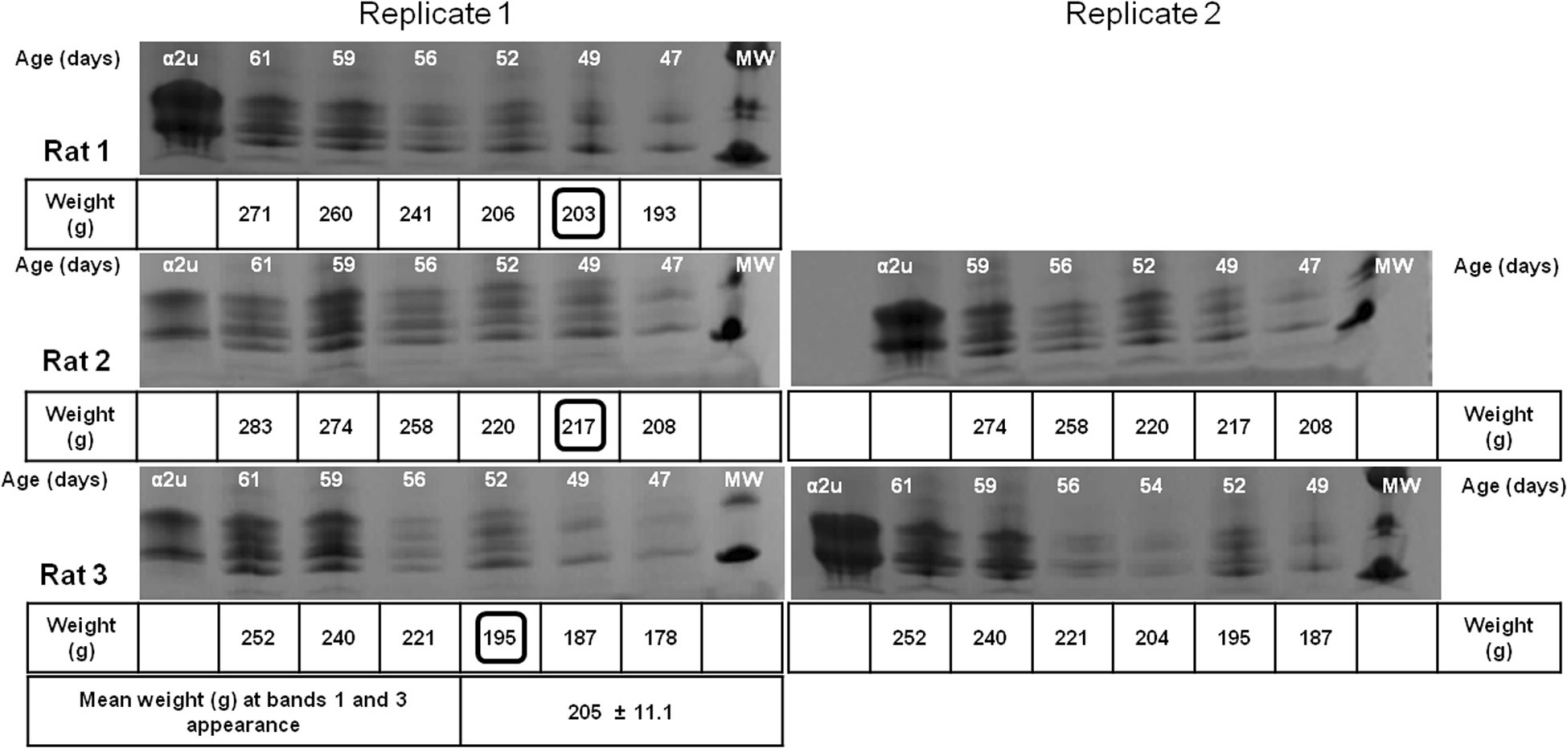
**Coomassie-stained SDS-PAGE (Phastgel gradient 10-15%) of urinary proteins of three different Sprague-Dawley male rats fed with maintenance diet.** Gel replicates of the same rats are shown (not for rat 1). The body weights of each animal at bands 1 and 3 first appearance have been highlighted with a square. The mean weight (± SD) of the animals when bands 1 and 3 appeared is also indicated. MW: molecular weight marker. α2u: α2u-globulin standard.

**Figure 4:**
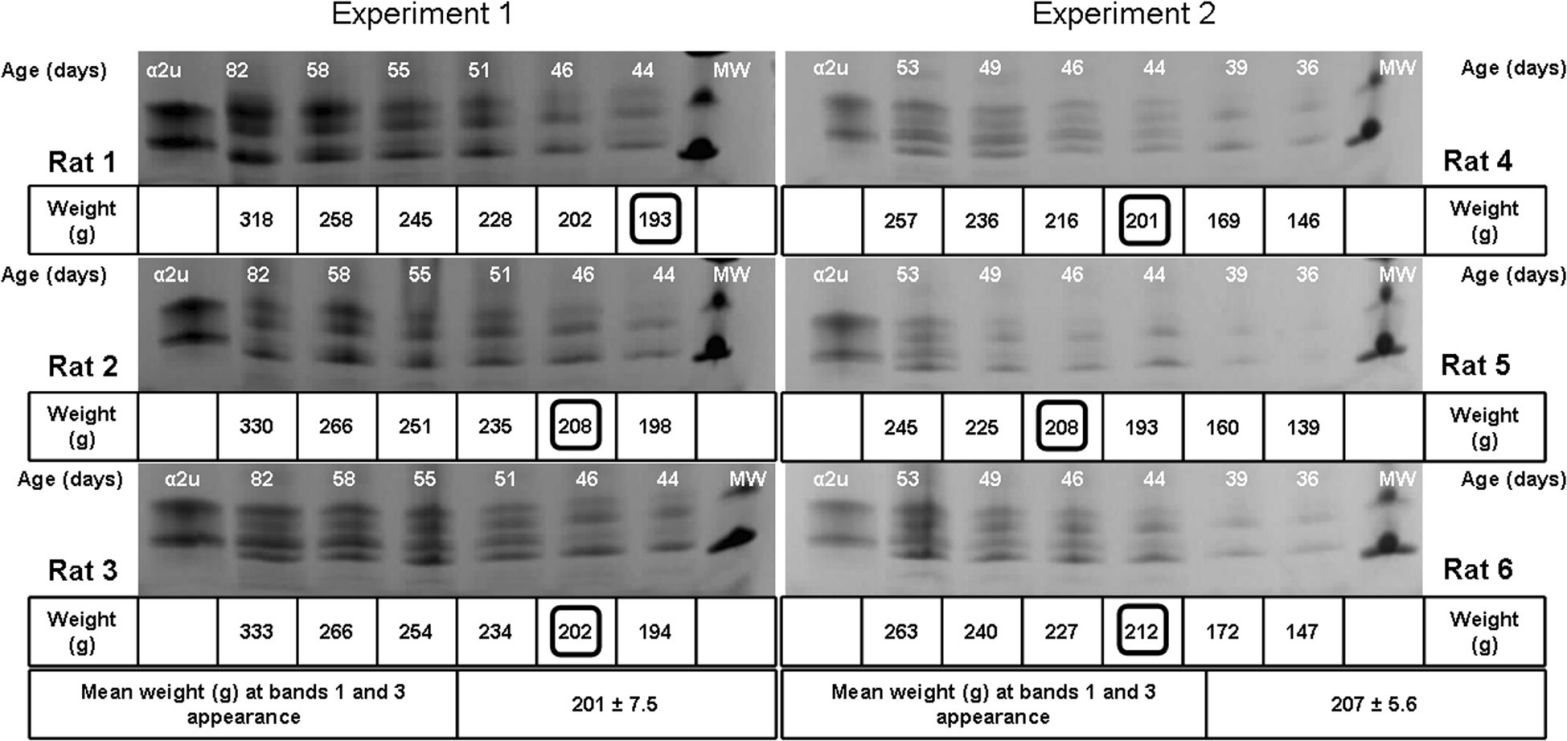
**Coomassie-stained SDS-PAGE (Phastgel gradient 10-15%) of urinary proteins of two independent experiments performed with Sprague-Dawley male rats fed with high protein diet (n = 3 rats per experiment).** The body weights of each animal at bands 1 and 3 first appearance have been highlighted with a square. The mean (± SD) animal weight at bands 1 and 3 first appearance is also indicated per experiment. MW: molecular weight marker. α2u: α2u globulin standard.

Gel electrophoresis revealed that the qualitative pattern of urinary proteins appeared generally to be similar in the two strains of rat (Figure 2A and B). The first-appearing electrophoretic bands were designated 2 and 4; thereafter a further pair of bands (1 and 3) of slightly higher MW appeared, which gradually became more prominent than bands 2 and 4, as implied from Coomassie staining intensity. Sprague-Dawley urines showed first occurrence of the full 4-band picture on maintenance diet at 49 or 52 days (Figure 3), but somewhat earlier on high protein diet at 44 or 46 days (Figures 2A and 4). All these stages corresponded to mean body weights of 205 g for Sprague-Dawley on maintenance diet and 201 g and 207 g for rats on high protein diets, the components of which clustered within the weight range 193-213 g. The more nutritious diet was therefore apparently reflected in faster progress towards a more complex pattern of lipocalins, even though body weight in the high protein group had not significantly diverged yet from that of the maintenance diet group.

**Figure 5:**
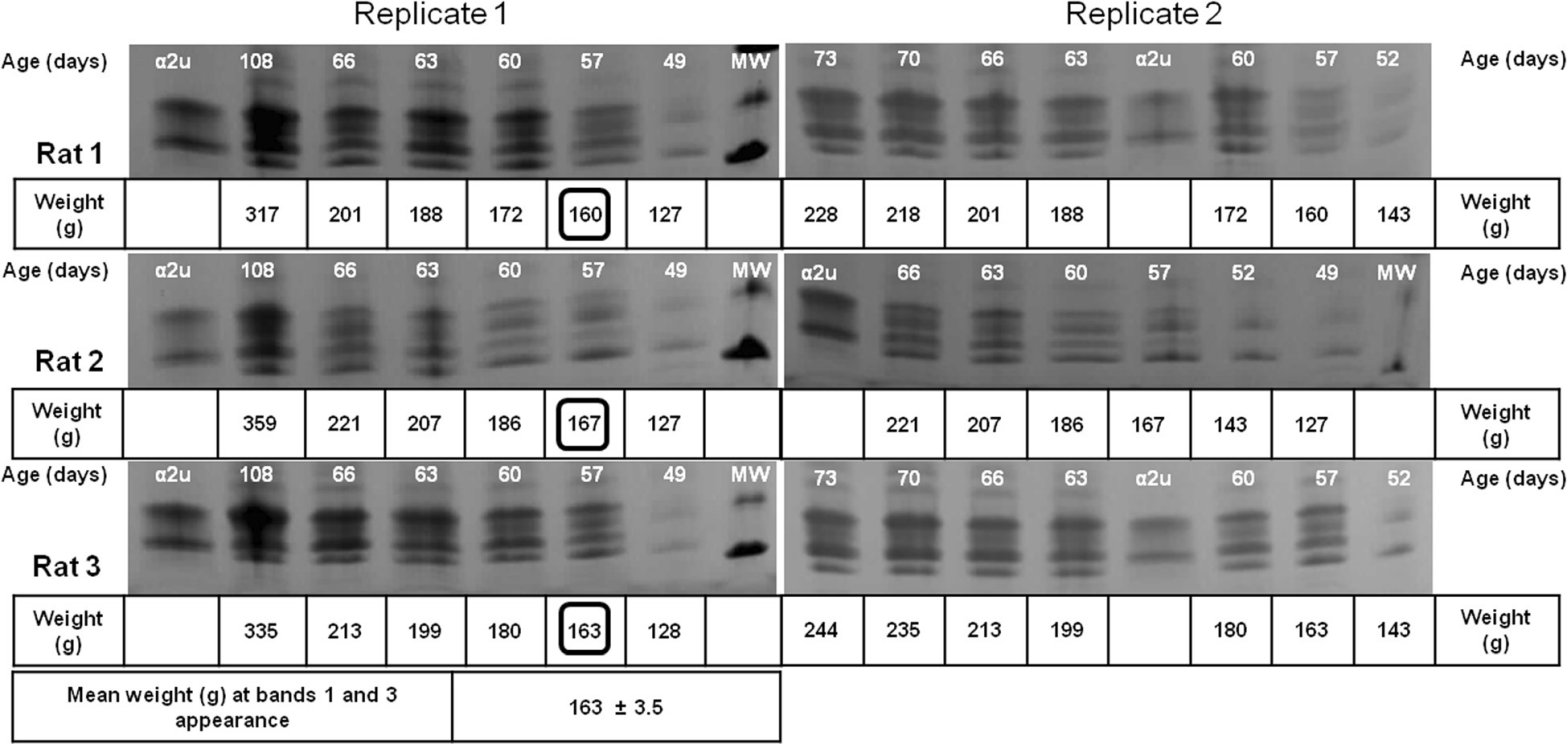
**Coomassie-stained SDS-PAGE (Phastgel gradient 10-15%) of urinary proteins of three different Wistar male rats fed with maintenance diet.** Gel replicates of the same rats are shown. The body weights of each animal at bands 1 and 3 first appearance have been highlighted with a square. The mean weight (± SD) of the animals when bands 1 and 3 appeared is also indicated. MW: molecular weight marker. α2u: α2u-globulin standard.

In contrast, in initially weight-matched rats of the smaller Wistar strain, with a slower growth rate than Sprague-Dawley animals also on maintenance diet (Figure 1), the lipocalin picture (Figures 2B, 5 and 10) changed later (by day 57) when only a much lower weight (163 ± 3.5 g) had been reached. Also in Wistars, bands 2 and 4 were still evident at 108 days. In both strains, electrophoretic mobility of bands 1 and 3 always corresponded to the two bands in the archived reference sample of α2u-globulin. In a Wistar rat at 57 days an albumin (SwissProt ALBU_RAT Accession number P02770), located near a 66 kDa marker (Figure 2B), was recognised from four tryptic digest peptide sequences (MASCOT score 153, not illustrated). This is not an unexpected finding; indeed a similar protein is increasingly apparent with age in the Sprague-Dawley gel (Figure 2A) and has been reported recently as normal in this strain [19].

**Figure 6:**
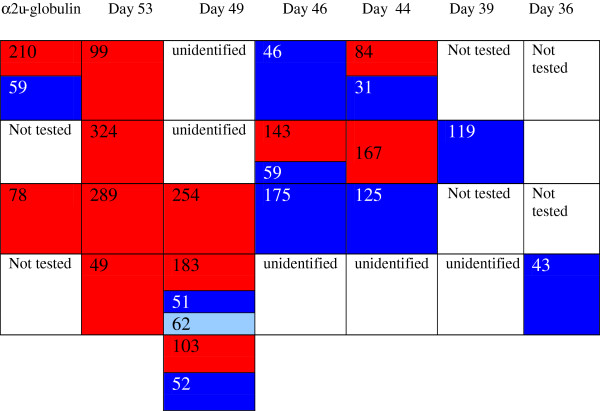
**Coded illustration of changes in composition of ~ 18 kDa urinary proteins during early puberty in Sprague-Dawley male rats on high protein diet, represented in Figure **2A**.** identified proteins in this gel. Red, Major urinary protein; Dark blue, Rat urinary protein 1; Light blue, Rat urinary protein 2. Blank spaces indicate either no obvious band, or that no identification from tryptic digest analysis was obtained. Numbers refer to MASCOT score.

**Figure 7:**
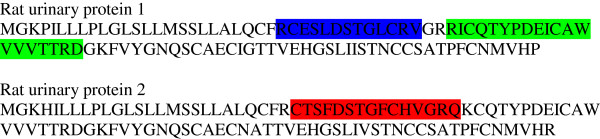
**Amino acid sequence of Rat urinary proteins 1 and 2 with matching highlighted sequences recognised in tryptic digest peptide sequences **[37][38]**.**

**Figure 8:**
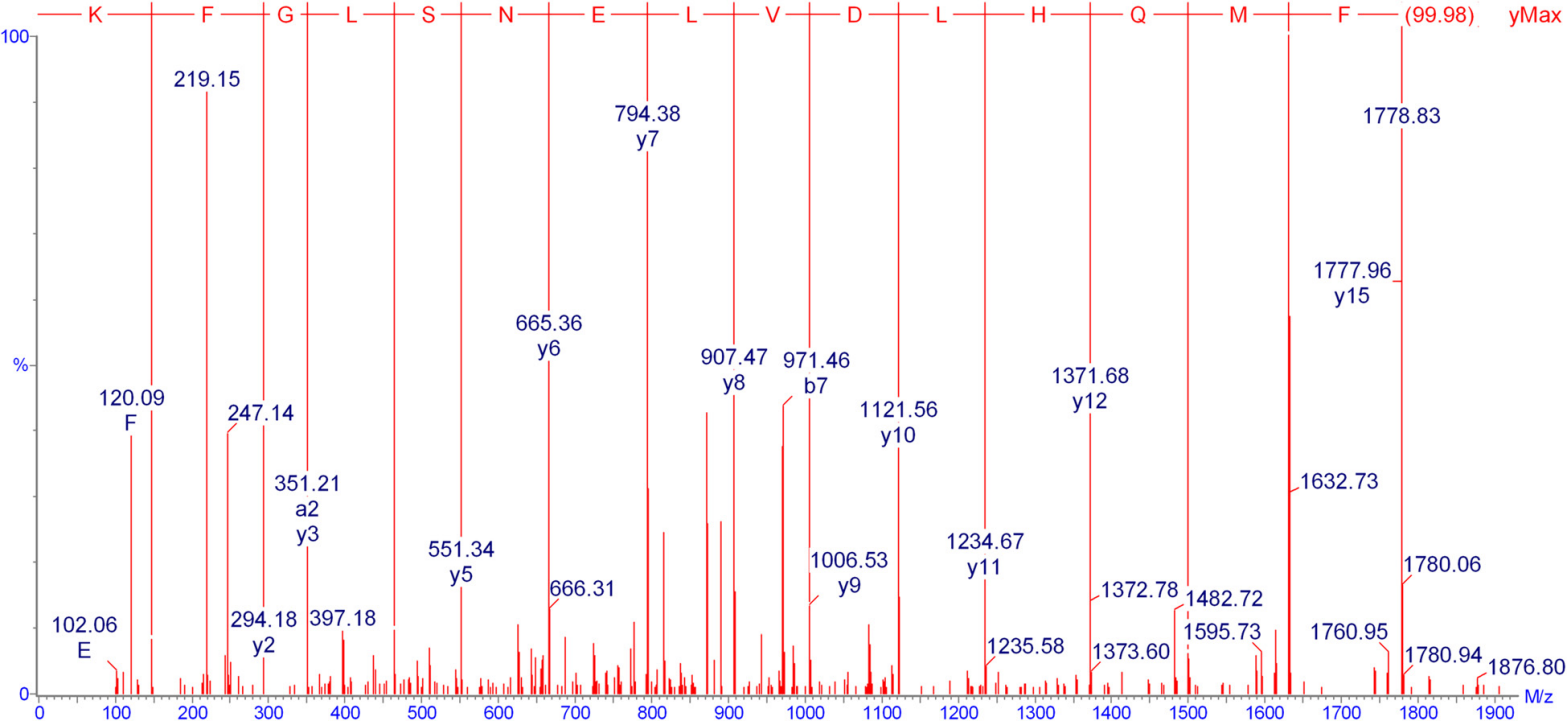
Product ion spectrum of the triply protonated peptide of m/z 626.3 corresponding to the sequence VFMQHIDVLENSLGFK from MUP_RAT.

**Figure 9:**
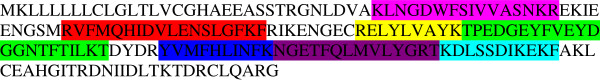
**Amino acid sequence of rat major urinary protein (α2u-globulin) with matching highlighted sequences recognised in tryptic digest peptide sequences.** Colouring of peptide sequences does not correlate with that in Figure 6.

Tryptic digest peptide sequence data was obtained for many of the Coomassie-stained bands in a gel electrophoretogram, resolved for urine of a Sprague-Dawley rat at intervals from 36-53 days of age (Figure 6). Figure 6 illustrates all the currently-identified lipocalins. At < 40 days old the digest data revealed two fragments with sequence fitting into the linear amino acid sequence of rat urinary protein 1 (Figure 7), and this protein was evident up to day 49. Rat urinary protein 2 (Figure 7) was also recognised on day 49 in this early puberty phase. Concurrently, the classic rat α2u-globulin, currently designated ‘major urinary protein’, appeared at day 44 (Figure 6), becoming dominant in the four bands which are the subject of the present focus by day 53. Sequences of all seven peptide fragments (Figures 8 and 9) found in various permutations of a maximum of five fragments in the gel bands fitted the published data for the 181-residue α2u-globulin (SwissProt MUP_RAT; Accession number P02761), illustrated in Figure 9. The sequence R.VFMQHIDVLENSLGFK.F was invariably present, and was also in bands of the α2u-globulin standard. Similar digest findings were evident in bands from a gel resolving some Wistar urines (Figures 2B and 5).

**Figure 10:**
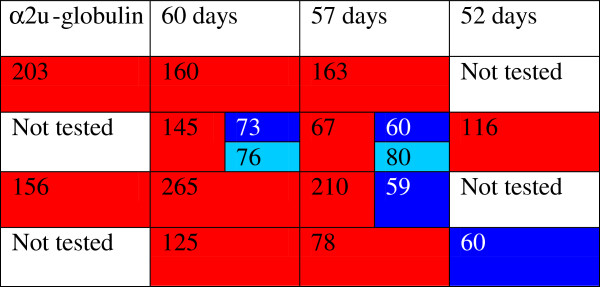
**Coded location of lipocalins in Wistar rat urine shown in Figure **2**B in the right hand four tracks showing temporal transition from rat urinary protein 1 to dominant α2u-globulin (matching the standard in track 4).** Numbers relate to MASCOT score.

**Figure 11:**
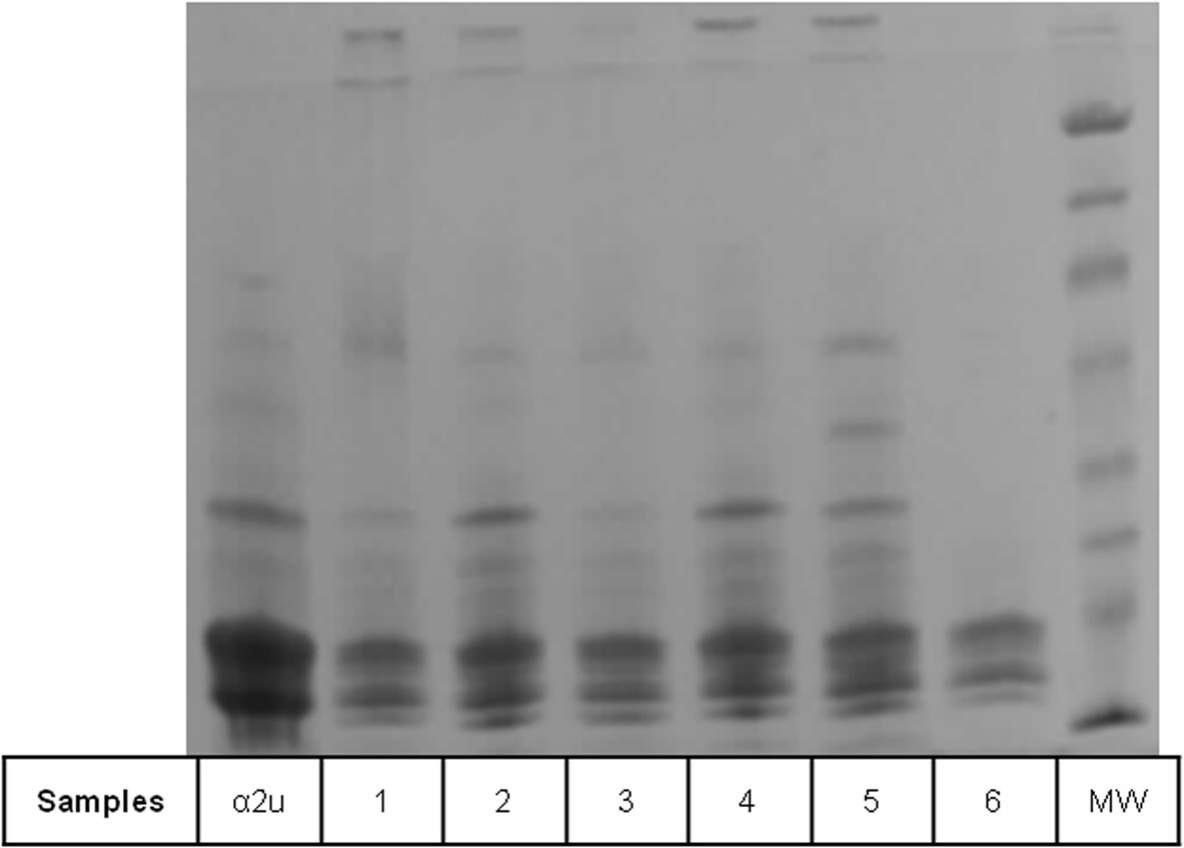
**Coomassie-stained SDS-PAGE (Phastgel gradient 10-15%) of urinary proteins of six different F 344 male adult rats (15 weeks old) fed with normal diet.** α2u: α2u-globulin standard.

Notably, rat urinary protein 1 was recognised (Figure 7) in one of the isoforms of the reference sample of α2u-globulin used in the gel shown in Figure 2A. Failure to recognise this complexity in the gel of Figures 2B and 10 may be caused by application of a smaller amount of standard. Reference sample occurrence of α2u-globulin implies that it had persisted as a minor hepatic metabolite, probably at least into adulthood. Concerning urine of several adult Fischer rats, the lipocalin pattern (Figure 11) in mini-gels was indistinguishable from findings in Sprague-Dawley and Wistar strains, but at present this does not necessarily imply that the constituent proteins are identical.

Several Wistar urine samples (Figure 5) that were also used for protein identification, provided data for testosterone and creatinine concentration (Table 1). Similar values recorded for 49 and 57 day old rats contrasted with higher well-replicated values at 107 days. When values are normalised against creatinine concentration, to account for possible idiosyncrasy in renal excretion, marked difference between animals at younger and older age was still apparent. Urines of the three Sprague-Dawley rats in the age range 36-56 days, given the high protein diet, were also analysed (Table 2). Normalised testosterone values varied over the period but also with no marked or consistent increase. Creatinine also varied over the period, occasionally up to two-fold. There could not have been any dilution of samples in the collection protocol, but surface evaporation in the cage could have imposed variable concentration.

**Table 1:** Urinary testosterone of Wistar male rats expressed adjusted according to associated creatinine concentration

**Wistar**	**Days old**								
		**49**			**57**			**107**	
**Rat**	**1**	**2**	**3**	**1**	**2**	**3**	**1**	**2**	**3**
Weight (*g*)	127	127	128	160	167	163	317	359	335
Testosterone	0.71	1.43	1.07	0.62	1.16	0.8	6.83	6.35	6.32
*(ng/ml)*									
Creatinine	61.4	68.2	72	40.5	89.3	119.1	157.4	167.1	196.4
*(mg/dL)*									
Testosterone/	1.16	2.10	1.49	1.53	1.3	0.67	4.34	3.80	3.22
Creatinine									
*(ng/mg)*									
**Mean**	**1.58**			**1.17**			**3.79**		
**( ± SD)**	**(0.48)**			**(0.45)**			**(0.56)**		

**Table 2:** **Urinary testosterone of Sprague-Dawley male rats (rats 4, 5 and 6 from Figure **4**) expressed adjusted according to associated creatinine concentration**

**SD high protein**	**Days old**						
	**36**	**39**	**44**	**46**	**49**	**53**	**56**
**Rat 4**							
Weight (*g*)	146	169	201	216	236	257	268
Testosterone (*ng/mL*)	1.4	2.77	1.16	2.73	2.72	2.41	1.53
Creatinine (*mg/dL*)	117.3	136.9	49.4	58.6	102.7	94	98.3
Testosterone/creatinine (*ng/mg*)	1.29	2.02	2.35	4.66	2.65	2.56	1.56
**Rat 5**							
Weight (*g*)	139	160	193	208	225	245	260
Testosterone (*ng/mL*)	0.64	0.54	2.1	1.57	1.86	1.63	1.58
Creatinine (*mg/dL*)	56.7	52.8	109.2	84.4	97.6	92.3	100.2
Testosterone/creatinine (*ng/mg*)	1.13	1.02	1.92	1.86	1.91	1.17	1.58
**Rat 6**							
Weight (*g*)	147	172	212	227	240	263	277
Testosterone (*ng/mL*)	1.68	1.15	2.16	3.91	3.59	5.99	2.95
Creatinine (*mg/dL*)	111.7	96.1	132.5	143.8	151	263.3	163.6
Testosterone/creatinine (*ng/mg*)	1.50	1.20	1.63	2.72	2.38	2.27	1.80

A 10% volume of 1% sodium azide solution was added to each collected urine sample as preservative. Values in the Table have not been adjusted to account for this relatively negligible dilution.

## Discussion

Excision of each band in the present gels sought to make a balance between both comprehensive recognition and demonstration of the principal component(s). Close electrophoretic mobilities of all these proteins means that examples could have appeared in one band in one time-sample and in an adjacent one in another.

Nevertheless, resolution of several rat urinary proteins in the 18 kDa range, some of which appear identical, in PhastSystem mini-gels still leaves open the possibility of a range of post-translational forms [20] which could include glycosylation. Use of a high protein diet in young rats did not appear to result in any marked change in urinary protein pattern but was associated with slightly earlier appearance of a 4-band lipocalin pattern. However, more abundant dietary protein might have diminished lipocalin salvage efficiency within nephrons, reflected in a greater abundance of the proteins in urine.

Considering the classical literature on α2u-globulin, comparison of its tissue-specific RNA from liver and both salivary and lachrymal glands in Sprague-Dawley rats Gubits et al. [15] found the RNA for the latter pair already in both sexes at 6 days old, whereas only at 44 days in liver. Kulkarni et al., from the same laboratory [21], also reported that 44 days was the stage at which a significant α2u-globulin transcription rate became evident. Also, administration (i.p.) of 17β-estradiol daily to male rats virtually eliminated hepatic α2u-globulin transcription within 8 days. Dot blot analysis of liver RNA from the same rat strain via hybridisation to nick-translated α2u-globulin cDNA gave no signal at 34 days of age, but a positive signal at and after 44 days. Findings in the present study (Figure 3) are consistent with this, but revealed other rat lipocalins in urine from 36 days old. Clearly there is much to understand about regulation of lipocalin synthesis in juveniles.

Roy et al. [11] recognised an iso-electric form of α2u-globulin by radioimmunoassay as early as 40 days old in the liver of Fischer rats. It is tempting to equate this with the ‘rat urinary protein’ observed first here in Sprague-Dawley rats, but Roy et al. [11] regarded this as the principal α2u-globulin. However, we presently resolve this into two forms. These may equate to forms A and B, previously recognised [13]. According to Roy et al. [5], maximum incidence of this compound occurred at 70 days, declining in later life. Otherwise it is difficult to equate any of the present urinary compounds defined by their amino acid sequence data with previous literature findings. This is partly due to the good resolution achieved in the PhastGels, but of course urinary content is only what remains after some of the glomerular-filterable lipocalins in blood plasma have been absorbed by proximal nephron epithelia for salvage of the nitrogen component. Nevertheless, non-invasive sampling gave insight into qualitative changes in circulating composition of lipocalins during early puberty and also demonstrated subsequent increased abundance of the two forms of these proteins in the ~18 kDa range. Relative molecular masses, assessed electrophoretically, were quoted as 18,800 and 18,100 [13], which could be accommodated in parts of the peptide sequence in Figure 8 that are unrecognised in the present tryptic digest protocol. Alternatively, post-translational modifications alone may accounts for the slightly different electrophoretic mobilities.

Biological significance of rat urinary proteins 1 and 2 is not yet known. However, recognition of rat urinary protein 1 in our reference sample of adult rat α2u-globulin, isolated originally at UK’s Imperial Chemical Industries (interested in light hydrocarbon nephropathy but before any biological function was known) in the 1980s [22], links the present study into a historical toxicology context and implies the former’s persistence as a minor urinary protein at least into adult (12-14 week old) life. Notably in [22], three isoforms of a generic α2u-globulin were recognised in 2D-PAGE of liver extracts of 32 outbred Wistar-derived rats. One isoform was common to all, but three combinations were recognised. One of these pairs characterised liver of 8 inbred F344 rats. However, pooled urine from Wistars contained all three α2u-globulin isoforms. From the findings of Elliott et al. [22] assumption of similarity of urinary lipocalin pattern of F344 rats in the present study with the other outbred strains requires structural conformation. Much remains to be understood.

Similar testosterone concentration, expressed relative to excretion of creatinine, on days 49 and 57 in Wistar males, and over the 36-56 day period in Sprague-Dawley animals on a high protein diet, demonstrated circulation of androgen already in the youngest animals. Over these periods there appeared to be a marked change in urinary protein composition, switching from rat urinary proteins alone to including Major rat urinary protein (Figures 3 and 5). This suggests that the latter’s appearance was not driven directly by a concentration surge of circulating androgen. However, apparent increase in overall abundance of urinary proteins by day 107 was clearly concomitant with the several-fold increase in circulating testosterone, if urine evidence at least roughly reflects the situation in its blood plasma source. Nevertheless, the present urine values of ~ 6 ng/ml are close to the 4 ng/ml reported for whole blood by radioimmunoassay in 6 month old Dark Agouti, Wistar and Sprague-Dawley males [23], and ~ 5 ng/ml, also by radioimmunoassay, in adult Sprague-Dawley rat serum [24]. However, we recognise that any further monitoring of circulating testosterone, particularly in pre-pubertal rats, should optimise urine sample collection to avoid any evaporation.

Although androgen-dependence as a classical descriptor for rat α2u-globulin is not questioned, the context and mechanism for this is unclear. In a recent publication [25] serum α2u-globulin concentration was measured (~ 50 μg/ml) 24 days after surgical castration, but unfortunately a value for entire rats at the same stage was not given. However, it was implied that although marked endocrine disruption for this lipocalin had occurred, it had not been complete. We deduce that androgen synthesis in adrenal glands may have sustained some hepatic α2u-globulin synthesis. Notably in the same context, daily sub-cutaneous testosterone replacement (0.5 mg/kg b.w.) was necessary significantly to increase α2u-globulin circulation 4-fold for similar castrates. Although sub-cutaneous, this seems a rather high testosterone dosage to equate approximately to the present 6 ng/ml urinary testosterone in Wistar males (Table 1, estimated ~ 60 ng excreted daily) that was adequate for maintaining abundant urinary α2u-globulin. Again, there is much to understand about androgen influence in rat hepatic α2u-globulin synthesis and in urinary lipocalins in general.

It is reasonable to perceive that, since steady-state plasma concentration of the xenobiotic ochratoxin A in female rats seems to be significantly higher than in males given equivalent chronic dietary doses [26], experimentally-impaired hormonal maleness in males could reduce this gender difference. Therefore, we cannot ignore findings of ongoing studies concerning gender-related differential toxicological responses of rats to chronic dietary exposure to ochratoxin A in which experimental interventions that markedly diminish circulating testosterone closed the gap between male and female titres for ochratoxin A in plasma as male values increased. Hepatic expression of α2u-globulin synthesis in rats is strictly androgen-dependent but is strongly suppressed by oestrogen [27]. Concurrent with the present study, the immunoblotting protocol of Mantle and Nagy [17] was revisited by us, although necessarily using a different ochratoxin A antibody, also sourced from R-Biopharm Rhone Ltd., Glasgow, UK. However, binding of ochratoxin A to α2u–globulin could not be confirmed. Both antibodies had been produced commercially specifically for the production of immuno-affinity columns, initially sourced from animals but latterly replaced by one from fermentation. Although when immobilised on the immunoaffinity column’s support substrate both were very efficient for selectively binding ochratoxin A, there could have been no expectation that the second antibody as a free protein would have had a binding domain for α2u–globulin, as had been apparent concerning the first-used antibody. Therefore, at present it has neither been possible to confirm nor to disprove the findings and perceptions of Mantle and Nagy [17]. However, favourable competition in circulating blood between a small lipocalin and serum albumin in binding ochratoxin A in males could provide a mechanism for maintaining a lower circulating OTA concentration than in comparable females. If a component of the α2u-globulin complex binds ochratoxin A this would be in dynamic competition with the well-known binding to serum albumin, and the ligand would easily take some toxin out of circulation with each pass through glomeruli in males and excrete via nephrons. Of course there would initially have been an interesting brief competition between small and large proteins in the hepatic portal vein for newly-absorbed ochratoxin A molecules before meeting newly-elaborated α2u-globulins in liver. Thus, a better understanding of the identity and kinetics of urinary lipocalins from early puberty seems to be required. Putative binding of ochratoxin A to a rat lipocalin now requires verification, hopefully *via* selection of a suitable ochratoxin A antibody from the many products available commercially. The structural conformation of this antibody must be able to retain its binding capacity to ochratoxin A even when part of the latter is located in a pocket domain of a lipocalin such as an α2u-globulin. Unfortunately the original antibody used by Mantle and Nagy [17] no longer exists.

We also note that, concerning the toxicological example of ochratoxin A discussed above, the immunoblot findings [17] were observed in urine of male F344 and F344 x Sprague-Dawley F_1_ hybrid rats. Earlier, F344 rats differed qualitatively in ‘α2u-globulin isoform’ composition from that of Sprague-Dawley or Wistar-derived strains [22]. F344 rats are favoured for some toxicology studies because they tend to reveal a worst-case scenario and were used in the 1989 US National Toxicology Programme study [28] revealing striking renal tumourigenesis in males in response to lifetime exposure to ochratoxin A, and in other studies cited in [17]. Wistar and Sprague-Dawley rats have not been used for lifetime renal tumourigenesis experiments with ochratoxin A. Therefore a question arises as to whether the immunoblot findings [17] were influenced by an F344 genome specificity for circulating lipocalins and might make F344 responses atypical for all rats. Unquestioning extrapolation from F344 responses to humans could then be unsafe.

## Conclusions

Convenience collection of small urine samples proved to be an ethical, economic, effective and non-intrusive source for monitoring dynamics both of small proteins and testosterone in growing rats. For the proteins, PhastGel separation seems to be method of choice. The pilot study allowed us to conclude that the qualitative change in urinary protein composition is probably reflective of similar temporal dynamics in hepatic synthesis and in blood. However, dynamics in blood would be much more difficult to observe in plasma because of its greater complexity. Consequently, resolution of major rat protein (α2u-globulin) into two components raises a question whether potential for binding of xenobiotics applies to both forms.

Whereas PhastGel separation of small urinary proteins already revealed complexity, further development of gradient separation should allow refined trypsin digest data on the homogeneity of bands. The present gels were a technical challenge in excision of narrow bands, and we chose examples with minimal amounts of proteins. Other significant peptide fragment patterns, attributable to as yet unrecognised proteins, could be revealed from study of less well resolved bands containing more protein. We conclude that proteomics may reveal a richer array of rodent urinary proteins than previously thought.

Expanded understanding of the dynamics of lipocalin synthesis, circulation, excretion and occurrence in urine, not only as young males enter puberty but also through adult life, has potential in explaining marked gender difference in response to some xenobiotic toxins where the mechanism yet remains obscure. We conclude that such potential should be explored further.

## Methods

### Animal experiments

Young male Wistar and Sprague-Dawley rats were housed in groups of three with pelleted feed (SDS 1 [maintenance, 14% protein] or SDS 3 [breeder, 21% protein], Special Diets Services, Essex, UK) and water *ad libitum* in an environment previously described [29]. Weight was recorded three times per week. For collection of urine for electrophoresis, also three times per week and with minimal animal disturbance, subjects were transferred individually on to the steel grid insert within a sterilised plastic mouse cage, without feed or water, for ~ two hours, during which a few drops of urine were released voluntarily on to the cage floor. Urine collection commenced in late morning, with the prospect of sufficient time for urination (~100 - 200 μl, then frozen at -20°C). The collection coincided with maximum blood concentration of testosterone expected during the light-phase part of the circadian cycle [30], so that testosterone and creatinine could be measured in some of the stress-free urine samples, even though these compounds are conventionally measured in blood [31,32]. All handling and procedures were carried out in accordance with the UK Animals (Scientific Procedures) Act 1986.

Urine of adult male Fischer rats from another study [33] was also used.

The same historic reference sample of rat α2u-globulin as used by Mantle and Nagy [17] was applied in this study.

### PhastGel electrophoresis of urine

The α2u-globulin analysis was performed using PhastSystem™ (Amersham Biosciences, Little Chalfont, Buckinghamshire, UK, HP7 9NA) and Phastgel Gradient 10-15% precast gels (43 × 50 × 0.45 mm) (GE Healthcare Bio-Sciences, Uppsala, Sweden), designed for 8 lanes. The urine samples were centrifuged for 5 min at 14000 g to remove particulate impurities. Then, 5 μl of urine was diluted with an equal volume of loading buffer and heated in a thermoblock at 88°C for 5 min. The α2u-globulin control was diluted ten-fold with loading buffer and heated similarly. Then, according to the manufacturer’s protocol, 1 μl of each sample was loaded on the precast gradient 10-15% gel and run for 20 min with Phastgel SDS buffer strips (GE Healthcare Bio-Sciences, Uppsala, Sweden). Gels were stained with Instant Blue^®^ (Expedeon, Babraham, UK) and the image was captured by a Syngene (Cambridge, UK) Diversity imaging system. Gels were calibrated by use of a low molecular mass ladder: 14.4, 20.1, 30, 45, 66, 97 kDa (Amersham LMW-SDS marker kit, GE Healthcare, UK).

### Characterisation of electrophoretically-resolved urinary proteins by LC-MS of tryptic digests

Selection of the gel for protein characterisations was made according to suitability for excision of bands with optimum resolution from adjacent components, particularly at the earliest stage of puberty. Thus the early growth stages of a Sprague-Dawley male on high protein diet, and a Wistar on maintenance diet, became the subjects of the pilot exploration of urinary protein changes.

### Protein identification by tandem mass spectrometry

Coomassie-stained gel bands were excised with a scalpel, and proteins were digested in gel with trypsin (specificity arginine and lysine), using an Investigator Progest robot (Genomic Solutions, Huntingdon, UK) as previously described [34].

Samples were analysed by high performance liquid chromatography coupled to electrospray ionisation tandem mass spectrometry (HPLC ESI MS/MS). HPLC was carried out on a CapLC liquid chromatography system (Waters, Manchester UK). Aliquots (6 μL) of peptide mixtures were injected onto a Pepmap C18 column (300 μm × 0.5 cm; LC Packings, Amsterdam, The Netherlands) and eluted with an acetonitrile/0.1% formic acid gradient to the nanoelectrospray source of a Q-Tof spectrometer (Micromass, Manchester, UK) at a flow rate of 1 μ/min. The spray voltage was set to 3500 V and data dependent MS/MS acquisitions were performed on precursor peptides with charge states 2, 3, or 4, over a survey mass range 440-1400, using argon collision gas. Product ion spectra were recorded over the range 100-1800, transformed onto a singly-charged *m/z* axis using a maximum entropy method (MaxEnt3, Waters, UK), and centroided peaklist (pkl) files were extracted using the MassLynx routine peptide auto (Waters, Manchester UK).

Proteins were identified by correlation of uninterpreted spectra to entries in SwissProt Release 2012_09 (538,010 entries) using a local installation of Mascot (version 2.2: http://www.matrixscience.com). MS/MS ion searches specified up to two missed cleavages per peptide, a precursor mass tolerance of ± 100 ppm and a fragment ion mass tolerance of ± 0.5 Da. Carbamidomethylation of cysteines and methionine oxidation were specified as fixed and variable modifications respectively.

### Criteria for protein identification

MS/MS based peptide and protein identifications were validated using Scaffold (Proteome Software Inc., Portland, Oregon: version 3.01) Peptide identifications were accepted if they could be established at greater than 95.0% probability as specified by the Peptide Prophet algorithm [35]. Protein identifications were accepted if established at greater than 99.0% probability and contained at least 2 matched peptides. Protein probabilities were assigned by the Protein Prophet algorithm [36].

### Measurement of testosterone and creatinine in urine

Urinary total testosterone concentration was measured by immunoassay with chemiluminescent detection following the automated Siemens method for Immulite 2500 Analyzer and creatinine by automated urinalysis in the University Clinic of Navarra, Pamplona, Spain.

## Competing interests

The authors declare that they have no competing interests.

## Authors’ contributions

PM, AV and JN developed the concept. PM and AV obtained animal samples. AV and JN obtained electrophoretic data. JM performed testosterone and creatinine measurements. RW obtained and interpreted proteomic data. PM wrote the paper in close collaboration with AV and RW. Authors have read and approved the final manuscript.
